# Mechanisms of Acquired Resistance to ALK Inhibitors and the Rationale for Treating ALK-positive Lung Cancer

**DOI:** 10.3390/cancers7020763

**Published:** 2015-04-30

**Authors:** Hideko Isozaki, Nagio Takigawa, Katsuyuki Kiura

**Affiliations:** 1 Department of Clinical Pharmaceutics, Okayama University Graduate School of Medicine, Dentistry, and Pharmaceutical Sciences, Okayama 700-8558, Japan; E-Mail: h.isozaki325@gmail.com; 2 Department of General Internal Medicine 4, Kawasaki Medical School, Okayama 700-8505, Japan; 3 Department of Allergy and Respiratory Medicine, Okayama University Hospital, Okayama 700-8558, Japan; E-Mail: kkiura@md.okayama-u.ac.jp

**Keywords:** lung cancer, EML4-ALK, crizotinib, ceritinib, alectinib, resistance

## Abstract

The discovery of an echinoderm microtubule-associated protein-like 4 (EML4)-anaplastic lymphoma kinase (ALK) fusion gene led to improved clinical outcomes in patients with lung cancer after the development of the first ALK-targeting agent, crizotinib. Some second-generation ALK tyrosine kinase inhibitors (TKIs), which might be more potent than crizotinib or effective on crizotinib-resistant patients, have been developed. Although these ALK-TKIs show an excellent response initially, most patients eventually acquire resistance. Therefore, careful consideration of the resistance mechanisms might lead to superior therapeutic strategies. Here, we summarize the history of ALK-TKIs and their underlying resistance mechanisms in both the preclinical and clinical settings. In addition, we discuss potential future treatment strategies in ALK-TKI-naïve and -resistant patients with lung cancer harboring the EML4-ALK fusion gene.

## 1. Introduction

Several gene aberrations are known to cause different types of cancer. This knowledge has led to the development of molecular-targeted drugs, and cancer treatment has progressed impressively over the past decade. The first important breakthrough was the development of imatinib, a BCR-ABL tyrosine kinase inhibitor (TKI), for chronic myeloid leukemia (CML) patients harboring the Philadelphia chromosome, which results from a translocation between chromosomes 9 and 22. This drug achieved long-term complete remission, which has excited physicians around the world. After 8 years of follow up, the complete cytogenetic response rate was 83%, and 55% of patients were still receiving imatinib. Only 7% of the patients died from CML, and the overall survival (OS) rate was 85% [1,2]. Subsequently, understanding the correlation between a mutation in the epidermal growth factor receptor (EGFR) gene and the response to gefitinib improved treatment strategies for advanced non-small cell lung cancer (NSCLC) [3,4]. Maemondo *et al*. reported that treatment with gefitinib achieved a longer median progression-free survival (PFS) of 10.8 months and a higher response rate of 73.7% compared with standard chemotherapy in NSCLC patients with *EGFR* mutations [5]. Following the success of EGFR TKIs, the discovery of the echinoderm microtubule-associated protein-like 4 (EML4)-anaplastic lymphoma kinase (ALK) fusion gene in 2007 elicited a significant change in the therapeutic strategies for NSCLC patients harboring the specific aberrant gene [6].

*ALK* is located on chromosome 2, and its gene product plays a role in brain development and acts on specific neurons in the nervous system [7]. An aberration in the ALK gene was reported first in patients with anaplastic large-cell lymphoma and inflammatory myofibroblastic tumors with an *ALK* translocation and *ALK* amplification, respectively [8,9]. In addition to the EML4-ALK fusion gene in lung cancer, various ALK-related diseases have been reported, including familial neuroblastoma [10], renal cell carcinomas [11,12,13,14], esophageal squamous cell carcinomas [15,16], breast cancer, colonic adenocarcinomas [17], glioblastoma multiforme [18,19], and anaplastic thyroid cancer [20]. These tumors were sensitive to ALK-TKIs [21,22]. Treatment with the first-generation ALK-TKI crizotinib exhibited prominent efficacy and became the standard therapy for NSCLC patients harboring the EML4-ALK fusion gene.

As stated above, the molecular-targeted agents identified to date have achieved outstanding efficacy. However, acquired (or initial) drug resistance is an inevitable problem. Herein we review the history of ALK-TKIs and their underlying resistance mechanisms. In addition, we summarize future therapeutic strategies for ALK-positive lung cancer patients.

## 2. Crizotinib

Crizotinib, a small molecule compound, can inhibit multiple tyrosine kinases and was developed initially for targeting MET. The EML4-ALK fusion gene was discovered as a novel driver oncogene in 2007 [6], and crizotinib has received attention, because it can also inhibit the ALK tyrosine kinase. Crizotinib is highly effective in patients with ALK-positive NSCLC, similarly to the effects of imatinib in BCR-ABL-positive CML or of gefitinib in EGFR-mutated NSCLC. It was reported that crizotinib achieved a longer PFS than did standard chemotherapy in advanced or metastatic ALK-positive NSCLC patients when used in both the first- and second-line settings (Table 1).

**Figure 1:**
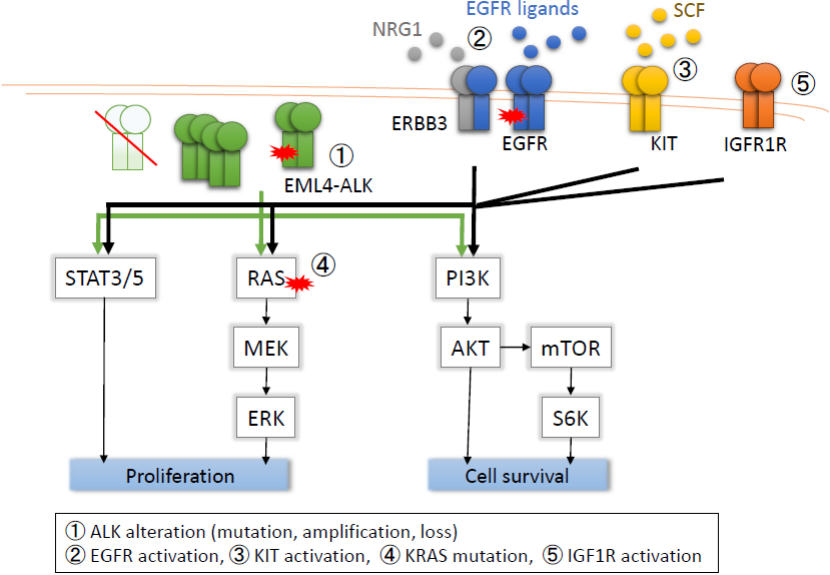
Mechanisms of resistance to crizotinib.

Although molecular-targeted compounds have elicited a good response in tumors expressing the specific target, crizotinib resistance also eventually occurs in almost all patients. The mechanisms of crizotinib resistance in patients and cell lines reported previously are summarized in Table 2 and Figure 1.

**Table 1:** Clinical Trials.

Drugs	Trial	Phase	Prior treatment with ALK-TKI	No. of patients	ORR	PFS	OS	CNS disease	Reference
Crizotinib	PROFILE 1001	1	No	143	60.8%	9.7 M	estimated 6 M: 87.9% 12 M: 74.8%		[23]
	PROFILE 1005	2	No (chemotherapy: Yes)	439	53%	8.5 M			[24]
	PROFILE 1007	3	No (platinum-based chemotherapy: Yes)	347 173 *vs*. 174	65% *vs*. 20%	7.7 *vs*. 3.0 M	12.2 *vs*. 12.1 M		[25]
	PROFILE 1014	3	No	343 172 *vs*. 171	74% *vs*. 45%	10.9 *vs*. 7.0 M	probability 12 M: 84% *vs*. 79%		[26]
Ceritinib	ASCEND-1	1	Yes (163/246)	246	58%	8.2 M	12 M: 65%	ORR: 54%	[27]
Alectinib	AF-001JP	1/2	No	Phase 1: 24 Phase 2: 46	93.5% CR rate: 19.6%	27.7 M	12 M: 83% 24 M: 79%		[28,29]
	AF-002JG	1/2	Yes	Phase 1: 47	55% CR rate: 2%	NA		ORR: 52%	[30]
AP26113	Gadgeel *et al*.	1/2	Yes	57	72%	10.9M		69% improved CNS disease	[31]

ORR: objective response rate; PFS: progression free survival; CNS: central nervous system; CR: complete response; M: month; TKI: tyrosine kinase inhibitor.

**Table 2:** Crizotinib resistance mechanisms.

Mechanisms			Material (patient or cell line)	Number of patients	Agents for overcoming the resistance		Reference
ALK alteration	ALK amplification	CNG	a patient	1 of 11 patients (12 samples)			[32]
	ALK amplification + ALK mutation	CNG + G1269A	a patient	1 of 11 patients (12 samples)			[32]
		CNG + L1196M	H3122CR1 cells (stepwise increase)		TAE684, AP26113, 17-AAG		[33]
		CNG + 1151Tins	H3122CR2 cells (stepwise increase)		17-AAG		[34]
	ALK mutation	L1196M, C1156Y	a patient	1			[35]
			Ba/F3 cells (transfected mutation)				
		F1174L	a patient	1			[36]
			Ba/F3 cells (transfected mutation)		TAE684, 17-AAG		
		S1206Y, G1202R, L1196M	patients	3 of 18 patients (19 samples)			[34]
		S1206Y, G1202R, L1196M, 1151Tins	Ba/F3 cells (transfected mutation)		S1206Y： TAE684, alectinib, 17-AAG; G1202R： TAE684, 17-AAG; L1196M： TAE684, 17-AAG, alectinib; 1151Tins： TAE684, 17-AAG, alectinib (Agents above showing lower IC_50_ than crizotinib)		[34]
		L1196M, G1269A	patients	4 of 11 patients (12 samples)			[32]
		G1202R	a patient	1			[37]
		L1152R	H3122 cells (transfected mutation)				[38]
Bypass track activation	ALK mutation + EGFR activation	L1152R	a patient	1		[38]	
		secretion of EGFR ligand (amphireglin)	DFCI076 cells (derived from the above-referenced patient)		ALK inhibitor + PF299804	[38]	
		1151Tins increased auto-phosphorylation of EGFR	a patient	1 of 18 patients (19 samples)		[34]	
	EGFR activation	L858R	a patient	1		[32]	
		retained phosphorylation of EGFR	H3122 cells (external EGF)		crizotinib + PF299804 or gefitinib	[38]	
			H2228 cells, H3122 cells (external EGF, TGF-α and HB-EGF)			[39]	
		secretion of EGFR ligand (amphiregulin) and ErbB3 ligand (NRG1)	H3122CR3 (stepwise increase)		crizotinib + gefitinib or erlotinib	[34]	
		increased auto-phosphorylation of EGFR	a patient	1 of 9 patients		[34]	
Bypass track activation	EGFR + KIT activation	Increased auto-phosphorylation of EGFR KIT amplification + SCF overexperssion	a patient	1 of 9 patients		[34]	
	KIT activation	KIT amplification	a patient	2 of 18 patients (19 samples)		[34]	
		increased phosphorylation of cKIT	H3122 (overexpressed cKIT + external SCF)		crizotinib + imatinib		
	KRAS mutation	G12V	patients	2 of 11 patients (12 samples) (1 of 2 is intrinsic resistances)		[32]	
			CUTO-1 cells (derived from above patient; ALK-, KRAS+)			[32]	
	IGF-1R activation	increased phosphorylation of IGF-1R	a patient	1		[37]	
			H3122 (external IGF-1R)		crizotinib + OSI-906	[37]	

CNG: Copy number gain; TAE684, alectinib: ALK inhibitor; 17-AAG: HSP90 inhibitor; PF299804: HER1, 2, and 4 inhibitor; OSI-906: IGF1R inhibitor; IC_50_: inhibitory concentration of 50%.

### 2.1. ALK Secondary Mutations

The genetic alterations underlying the acquired resistance to molecular-targeted agents such as T315I in BCR-ABL or T790M in EGFR have been reported previously [40,41,42]. Approximately 50% of NSCLC patients who acquire resistance to gefitinib exhibit secondary mutations. Among the reported mutations, >90% of the resistant tumors have a T790M gatekeeper mutation, which is present in the binding site for competitive inhibitors in the adenosine triphosphate (ATP)-binding pocket of various kinases [43]. Additional mutation sites in EGFR-TKI resistant tumors have been reported only rarely. In contrast, crizotinib-resistant ALK-positive NSCLC tumors have multiple gene alterations both inside and outside the gatekeeper site, similar to imatinib-resistant gene alterations.

**Figure 2:**
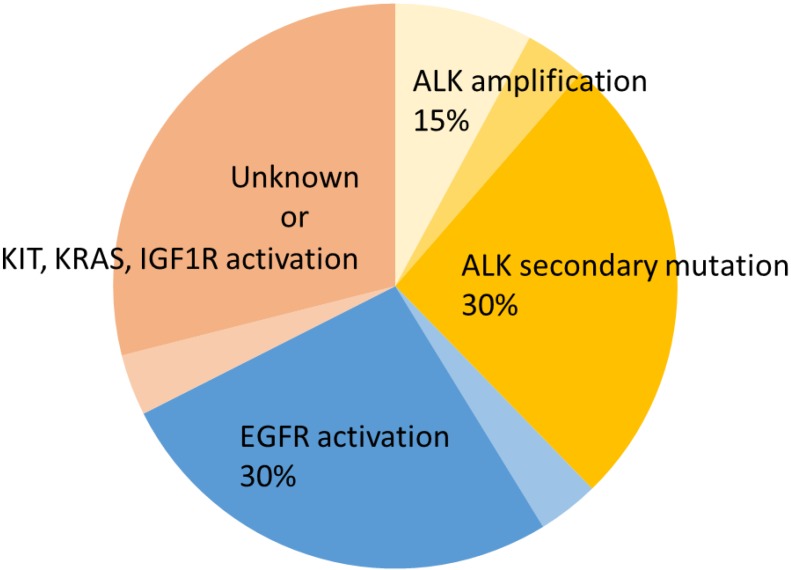
Approximate proportion of crizotinib-resistant mechanisms [32,34]. ALK secondary mutations include 1151Tins, L1152R, C1156Y, F1174L, L1196M, G1202R, S1206Y, and G1269A.

Katayama *et al*. performed a comprehensive investigation of re-biopsy samples derived from 18 crizotinib-refractory patients and found secondary ALK mutations (S1206Y, G1202R, L1196M, and 1151Tins) in four patients [34]. Doeble *et al*. also observed two different mutations (L1196M and G1269A) within the tyrosine kinase domain in four of nine patients with acquired resistance to crizotinib [32]. Based on the two reports described above, various ALK secondary mutations might occur in ~30% of crizotinib-refractory patients (Figure 2). Furthermore, multiple gene alterations were observed in one patient. Choi *et al*. described two different secondary mutations in the kinase domain of *EML4-ALK* in tumor cells isolated from a crizotinib-refractory patient [35], one of which was the gatekeeper mutation L1196M, which corresponds to T315I in BCR-ABL and T790M in EGFR. The other mutation was C1156Y, which is located within the ALK kinase domain N-terminal to the αC-helix. These results suggest that secondary alterations to ALK in crizotinib-resistant patients can present with heterogeneity, even within a single tumor.

Some second generation ALK inhibitors have been developed, including ceritinib and alectinib, which could overcome the ALK secondary mutations [44,45]. Tumors with different mutations exhibit different sensitivities to the agents. Thus, differing approaches according to the type of mutation are required to overcome crizotinib resistance, including ALK secondary mutations. To date, two ALK secondary mutations that seem to be refractory to ceritinib and/or alectinib have been reported: cells with the G1202R mutation were insensitive to ceritinib and alectinib, and cells with the F1174C mutation were insensitive to ceritinib [34,45,46]. The sensitivity of cells with F1174C mutations to alectinib has not been reported. The development of novel agents that are effective in NSCLC patients with the secondary mutations described above is expected in the near future.

### 2.2. ALK Amplification

A gain in ALK gene fusion copy number was implicated as a mechanism of resistance to crizotinib in a cell line model [33]. Cells resistant to an intermediate dose of crizotinib (0.6 μM) developed an amplification in the ALK gene, which was retained in cells resistant to a high dose (1 μM) of crizotinib. Interestingly, cells that were completely resistant also harbored the gatekeeper mutation L1196M, which was not detected in the partially resistant cells. Doeble *et al*. also reported an *ALK* copy number gain in two re-biopsied samples [32]. One sample had an *ALK* secondary mutation (G1269A) together with *ALK* amplification, and the other had no secondary mutation but possessed an *ALK* copy number gain of 2.2-fold, which was the highest gain among the 10 samples examined in the study. Thus, *ALK* secondary mutations might occur when ALK-positive tumors harboring a low level of *ALK* amplification acquire high crizotinib resistance. In addition, if *ALK* amplification in the absence of a secondary mutation causes crizotinib resistance, a high level of amplification may result in the resistant tumor. A preclinical study demonstrated that a second-generation ALK inhibitor or an HSP90 inhibitor might abrogate this resistance [33,34].

### 2.3. Loss of ALK

In addition to secondary *ALK* mutations and *ALK* amplification, the loss of *ALK* or other alterations in driver oncogenes were reported in crizotinib-resistant tumors. Two of nine ALK-positive NSCLC tumors lost the EML4-ALK fusion gene after treatment failure with crizotinib [32]. Of these, one sample showed loss of *ALK* in the first re-biopsy performed 61 days after the initiation of crizotinib treatment. However, the *ALK* fusion gene was re-detected in a second re-biopsy after 113 days on crizotinib. It is difficult to prove loss of *ALK* in clinical samples, because ALK detection is affected easily by various factors. In addition, we always have to consider whether the specimens contain sufficient cancer cells for analysis and whether the processes used to obtain formalin-fixed paraffin-embedded sections and store samples are appropriate [47]. A recent report demonstrated that loss of the ALK fusion gene occurred in one crizotinib-refractory NSCLC patient using next generation sequencing [48]. Nevertheless, future investigations using preclinical models and more clinical samples are needed to determine whether loss of ALK is a mechanism of resistance to crizotinib.

### 2.4. EGFR Activation

In addition to ALK gene alterations, other mechanisms of resistance to crizotinib include the activation of tyrosine kinase receptors such as EGFR, KRAS, or cKIT. Because these resistance mechanisms partially retain ALK survival signaling (namely the co-activation of ALK and other tyrosine kinase pathways), blocking both pathways is likely required to overcome this resistance. Sasaki *et al*. detected the secretion of amphiregulin, which is an EGFR ligand, in DFCI076 cells derived from a crizotinib-refractory patient [38]. DFCI076 cells had no *ALK* secondary mutations and displayed increased levels of activated EGFR compared with H3122 cells. They also confirmed that the presence of exogenous EGF caused crizotinib resistance in H3122 cells, suggesting that EGFR activation might be induced by increased EGFR ligand levels. Yamada *et al*. also revealed that paracrine EGFR ligands including EGF, TGF-α, and heparin-binding EGF-like growth factor (HB-EGF) caused crizotinib resistance [39]. Consistent with this phenomenon, Katayama *et al*. observed increased levels of *EGFR* mRNA, amphiregulin, and HER3 ligand (NRG1) in the resistant cells, in which activation of EGFR and HER3 was increased compared with the parental cells [34]. Four of nine crizotinib-resistant tumors expressed increased levels of phosphorylated EGFR compared with pre-resistant samples. In addition, a preclinical study showed that the combination of crizotinib and EGFR-TKIs might be effective against this resistance [34,38]. Katayama *et al*. found that NSCLC with an *ALK* secondary mutation (1151Tins) expressed activated EGFR [34]. Thus, the activation of alternative survival signaling pathways could coexist with *ALK* secondary mutations.

### 2.5. cKIT Amplification

Crizotinib resistance caused by amplification of the cKIT gene was reported in two samples derived from crizotinib-refractory patients [34]. One tumor expressed high levels of the KIT ligand, stem cell factor (SCF) in stromal cells within the solid component. H3122 cells overexpressing KIT were sensitive to crizotinib in the absence of exogenous SCF, although they remained resistant to crizotinib in the presence of SCF. Therefore, this type of resistance might require both activated tyrosine kinase receptor and presence of the ligand. Treatment with imatinib, a small molecule inhibitor of KIT, reversed the resistance phenotype completely, suggesting that imatinib is useful for treating KIT-overexpressing crizotinib-refractory NSCLC.

### 2.6. KRAS Mutation

Oncogenic mutations within the KRAS gene (G12C or G12V) were reported in two of 11 NSCLC patients [32]. However, given that one patient had a short duration to progression despite treatment with crizotinib and harbored a *KRAS* G12C mutation *ab initio*, this case was considered to be intrinsic resistance. The other patient exhibited a *KRAS* G12V mutation; however, introducing a *G12V* mutation into the *KRAS* gene did not elicit crizotinib resistance in H3122 cells. Thus, it remains unclear whether the acquisition of a *KRAS* mutation could serve as a direct mechanism of acquired resistance to crizotinib.

### 2.7. IGF-1R Activation

A previous study reported that expression of IGF-1R and insulin receptor substrate-1 (IRS-1), which is an adaptor protein that binds to IGF-1R or ALK, were related to crizotinib resistance [37]. Three tumor biopsies taken at the time of acquired resistance expressed increased levels of pIGF-1R compared with the respective pre-resistant tumors. Two of three samples had increased IRS-1 expression after crizotinib treatment compared with pretreatment. In one tumor, *IGF-1R* and *IRS-1* mRNA levels were increased after crizotinib treatment. The preclinical study suggested that the combination of crizotinib and an IGF-1R inhibitor might overcome this resistance. They also found that there was no significant change in IRS-1 levels between before and after EGFR-TKI therapy in *EGFR*-mutant lung tumor. Namely, the changes in IRS-1 might be specific to ALK-positive lung cancer.

## 3. Ceritinib

On April 29, 2014, the Food and Drug Administration (FDA) approved the use of ceritinib for the treatment of ALK-positive metastatic NSCLC patients who are intolerant to crizotinib or have progressed after crizotinib treatment. Ceritinib is a second-generation ALK-TKI that can block not only ALK, but also IGF-1R and INSR [37,49]. In addition, previously untreated patients with central nervous system (CNS) involvement also achieved a favorable response to ceritinib, with an overall response rate (ORR) of 72.3% and a PFS of 18.4 months [50]. In a phase 1 study of patients with advanced NSCLC harboring ALK rearrangements, ceritinib demonstrated good efficacy, with an ORR of 58% and a median PFS of 7.0 months [27]. Subgroup analyses revealed that ceritinib exhibited good activity in crizotinib-refractory patients (ORR, 56%) as well as in patients that had not received crizotinib (ORR, 62%). However, differences in the median PFS were observed among subgroups: the median PFS of crizotinib-refractory patients was 6.9 months, whereas the crizotinib-untreated group did not reach the median. Although ceritinib could salvage crizotinib-refractory patients temporally, recurrence was observed earlier in these individuals compared with crizotinib-naïve patients. Thus, studies of ceritinib resistance will be very important.

A preclinical study reported that ceritinib was sensitive to cell lines expressing ALK with ALK-TKIs-resistant mutations (L1196M, I1171T, S1206Y and G1269A), but could not inhibit the growth of cells with G1202R and F1174C mutations in *ALK* [46]. In a phase 1 study, *ALK* secondary mutations (1151Tins, L1196M or G1269A) were detected in refractory tumors after crizotinib-naïve patients were treated with ceritinib. These results suggest that ceritinib could overcome resistance by acquiring L1196M or G1269A mutations, whereas ceritinib might paradoxically cause these mutations. In addition, a preclinical study showed that ceritinib could overcome alectinib resistance, including in tumors with *ALK* secondary mutations (I1171T and V1180L) [51]. In contrast, preclinical studies demonstrated that the resistance induced by L1196M and G1269A mutations which could occur in patients with ceritinib-resistance could be overcome by alectinib [44,45]. Therefore, alectinib might be useful in ceritinib-refractory patients.

## 4. Alectinib

Alectinib is a selective and potent ALK inhibitor [44] that was approved in Japan on 4 July 2014. A recent study also revealed that the drug inhibited RET kinase [52]. In a Japanese phase 2 trial of ALK inhibitor-naïve patients with ALK-rearranged advanced NSCLC, alectinib achieved an excellent response rate of 93.5%, a long response duration, and an acceptable toxicity profile [28]. A total of 19.6% of patients achieved a complete response, and the 2-year progression-free survival rate was 76%. There was no progression in CNS lesions among patients with known CNS metastases at baseline [29]. A study performed in the United States in ALK-positive NSCLC patients treated previously with crizotinib (but not with other ALK inhibitors) demonstrated a good ORR of 54.5%. The preliminary results also suggested that alectinib might have antitumor activity against CNS lesions in patients with crizotinib-refractory disease [53]. Therefore, the FDA granted alectinib a “breakthrough therapy” designation for patients with metastatic NSCLC that progressed after treatment with crizotinib in 2013. A preclinical study demonstrated the efficacy of alectinib in intracranial metastases. Specifically, treating a mouse xenograft model formed from H2228 cells with alectinib resulted in regression of the brain tumors and provided a survival benefit. In a pharmacokinetic study in rats, alectinib achieved a high brain-to-plasma ratio [54]. Actually, some crizotinib-refractory patients with CNS metastasis had response to alectinib [55,56,57,58]. Alectinib also had substantial inhibitory potency against tumors with *ALK* secondary mutations (addition to gatekeeper mutation L1196M, 1151Tins, L1152R C1156Y, F1174L and G1269A). However, alectinib seems to be less potent against *ALK* G1202R mutations [45,59].

Katayama *et al*. discovered the V1180L *ALK* gatekeeper mutation *in vitro* and an I1171T *ALK* mutation in a tumor from a patient treated with crizotinib followed by alectinib. In another report, a patient with alectinib-resistance harbored the I1171T *ALK* mutation [60]. In addition, ALK-positive tumors harboring V1180L and I1171T mutations could be suppressed by the second-generation ALK inhibitors, ceritinib, AP26113, and ASP3026. Ceritinib and AP26113 were effective at inhibiting tumors expressing the V1180L and I1171T mutations, whereas ASP3026 seemed to be inactive against I1171T Ba/F3 murine cells [51]. As well as crizotinib-resistance, we would have to select the suitable drug in each alectinib-refractory patient with the *ALK* secondary mutation.

*MET* amplification was observed in alectinib-refractory tumors, although tumor samples from before the alectinib treatment were not available [31]. The patient experienced a dramatic response to crizotinib, which strongly inhibits MET tyrosine kinase. Moreover, MET activation induced by hepatocyte growth factor (HGF) in an autocrine manner was observed in an alectinib-resistant cell line [61]. The cells were sensitive to crizotinib both *in vitro* and *in vivo*. Thus, crizotinib might be effective against crizotinib-naïve tumors expressing MET activation after treatment with alectinib. Two randomized clinical trials comparing alectinib with crizotinib in the first-line setting are ongoing (J-ALEX and ALEX studies). In addition, a phase 2 study (UMIN000015984) of crizotinib monotherapy in patients with alectinib-refractory NSCLC harboring EML4-ALK would be interesting.

## 5. Other Novel ALK Inhibitors

In addition to ceritinib and alectinib, other second-generation ALK inhibitors (AP26113, ASP3026, TSR-011, PF-06463922, RXDX-101, X-376, X-396, CEP-28122, and CEP-37440) have been developed to date (Table 3) [62]. These new agents are expected to exhibit efficacy in the CNS and might help overcome drug resistance.

**Table 3:** ALK inhibitors.

Drugs	Company	Other activity	Clinical trials	Status
Crizotinib (PF-02341066)	Pfizer	MET, ROS1	Phase 1, 2, 3	Approved by FDA (Auguet 2011) Clinically available in Japan (March 2012)
Ceritinib (LDK378)	Novartis	IGF1R, INSR	Phase 1, 2, 3	Approved by FDA (May 2014)
Alectinib (CH5424802)	Chugai, Roche	RET	Phase 1, 2, 1/2, 3	Breakthrough Therapy Designation (June 2013) Clinically available in Japan (July 2014)
AP26113	Ariad	EGFR, ROS1	Phase 1/2	Breakthrough Therapy Designation (October 2014)
ASP3026	Astellas	ROS1	Phase 1	
X-376 X-396	Xcovery	MET	Phase 1 (X-396)	
TSR-011	Tesaro	TRK-A, TRK-B, TRK-C	Phase 1/2a	
RXDX-101	Ignyta	ROS1 TRK-A, TRK-B, TRK-C	Phase 1	
CEP-28122 CEP-37440	Teva	RSK2, RSK3, RSK4	Phase 1 (CEP-37440)	
PF-06463922	Pfizer	ROS1	Phase 1/2	

AP26113 is a potent TKI that can inhibit both ALK, EGFR and ROS1 [63]. In an ongoing phase 1/2 study of AP26113 (NCT01449461), patients with ALK-positive NSCLC resistant to crizotinib demonstrated an ORR of 67%. Four of five patients with ALK-positive CNS metastatic lesions experienced tumor regression [64]. In a preclinical study using Ba/F3 cell lines expressing clinically identified EML4-ALK mutations, AP26113 was effective on crizotinib- or alectinib-resistant mutations (L1196M, V1180L, and I1171T) [63].

A phase 1 study of ASP3026, which inhibits ALK, ROS1, ACK and EGFR *in vitro*, showed a favorable safety profile in patients with advanced solid tumors despite ALK positivity (NCT01401504). The tumor response has not been reported [65]. As described previously, ASP3026 was effective in tumors expressing resistance mutations, including the *ALK* gatekeeper mutations L1196M in crizotinib-resistant and V1180L in alectinib-resistant preclinical models [51].

PF-06463922 is a small ATP competitive inhibitor of ALK/ROS1 that penetrates the blood-brain barrier in preclinical animal models. Thus, it exhibits high activity in crizotinib-resistant cells [66]. A phase 1/2 study in patients with ALK-positive and ROS1-positive NSCLC is ongoing (NCT01970865).

X-376 and X-396, which suppress the kinase activity of ALK and MET, were developed. In contrast, crizotinib was shown to be a slightly more potent MET inhibitor than X-376 or X-396. In addition, X-396 could inhibit the crizotinib-resistant mutants L1196M and C1156Y potently [67]. Recently, mechanisms of resistance to X-376 were reported in a resistant cell line model (H3122 XR) established from parental H3122 cells. H3122 XR exhibited increased IGF-1R phosphorylation when IGF-1R was overexpressed. H3122 XR cells were sensitive to the combination of an IGF-1R inhibitor or anti-IGF-1R antibody and X-376 [37]. A phase 1 trial of X-396 is ongoing in patients with advanced solid tumors (NCT01625234).

TSR-011 is a potent ALK-TKI. Although a phase 1/2 study (NCT02048488) is ongoing, four of six evaluable ALK-positive and crizotinib-pretreated patients achieved a response. Interestingly, the drug exhibited activity against tropomyosin-related kinase (TRK) A, B, and C receptor (encoded by *NTRK1*, *NTRK2*, and *NTRK3*, respectively) both *in vitro* and *in vivo* [68]. Because the driver oncogene of NTRK1 in NSCLC has been reported [69], the effects of TSR-011 in NSCLC with TRK rearrangements should be explored further.

RXDX-101 also inhibits ALK, ROS1, TRK-A, TRK-B, and TRK-C. It is active against the *ALK* mutants responsible for crizotinib resistance (L1196M and C1156Y) and crosses the blood-brain barrier in a mouse brain metastatic model [70,71]. In an ongoing phase 1 study, NSCLC and neuroblastoma patients with *ALK* mutations, NSCLC patients with *ROS1* mutations, and colorectal cancer patients with *TRK-A* mutations achieved a response without dose-limiting toxicity (NCT02097810) [72].

CEP-28122 is a selective active ALK inhibitor with favorable pharmaceutical and pharmacokinetic profiles against ALK-positive tumors *in vitro* and *in vivo* [73]. Although it has not been in clinical development, a phase 1 trial (NCT01922752) is ongoing using CEP-37440, which inhibits both ALK and focal adhesion kinase (FAK); however, the details have not yet been reported.

## 6. Conclusions and Future Therapeutic Strategies

Since the FDA approved crizotinib in 2011, understanding of its efficacy, toxicity, and resistance has increased. Crizotinib is a key drug in the current therapeutic strategy for ALK-positive lung cancer. However, it has a limited response duration, and drug resistance must be considered. In addition, crizotinib has limited efficacy in the CNS. Therefore, second generation ALK-TKIs are required. Because there are a variety of crizotinib resistance mechanisms, various mechanisms should be assessed using resistant samples to provide appropriate treatment for patients.

The most common mechanism of crizotinib resistance is a series of *ALK* secondary mutations. Unlike gefitinib resistance, *ALK* secondary mutations are miscellaneous, because each mutation has a different sensitivity to different second-generation ALK inhibitors. If a crizotinib-refractory patient has the L1196M *ALK* gatekeeper mutation, alectinib or an HSP90 inhibitor might be effective. In contrast, ceritinib, alectinib, AP26113, or PF-06463922 might be preferred in crizotinib-refractory patients with brain metastasis. In cases of crizotinib resistance involving EGFR or IGF-1R, ASP3026 (a dual ALK/EGFR inhibitor) or ceritinib (which inhibits ALK and IGF-1R) would be reasonable.

Although crizotinib has achieved a higher response rate than chemotherapy in first- and second-line setting phase 3 trials, the OS between the crizotinib-arm and chemotherapy-arm are similar so far. In the second-line study, crizotinib was superior to chemotherapy (ORR, 65% *vs*. 20%, respectively; PFS, 7.7 months *vs*. 3.0 months) [25]. In the first line study, standard chemotherapy in *ALK*-positive patients achieved a good response (ORR, 74% in crizotinib-arm *vs*. 45% in chemotherapy-arm; PFS, 10.9 months *vs*. 7.0 months) [26]. The similar OS between the two arms might be a result of the effective treatment achieved using chemotherapy. Thus, advanced ALK-positive NSCLC patients should be treated with both ALK-TKIs and chemotherapy if they have a good performance status. Actually, there were so-called “super-responders” to pemetrexed among ALK-positive patients [25]. When a biomarker confirming the efficacy of pemetrexed in selected patients is identified and confirmed, chemotherapy based on precision medicine could be achieved.

Although treatment with crizotinib is standard today, alectinib is also likely to be administered in the first line setting. In the near future, crizotinib or ceritinib might be selected in alectinib-refractory patients with activated *MET* or secondary mutations (V1180L or I1171T). In addition, alectinib might be acceptable in frail patients who are elderly or have a poor performance status, because the adverse effects of alectinib seem to be milder compared with those associated with crizotinib.

On February 2, 2015, the anti-PD-L1 agent MPDL3280A received a breakthrough therapy designation from the FDA as a potential treatment for patients with PD-L1-positive NSCLC who progressed on platinum-based chemotherapy and an EGFR or ALK inhibitor. This suggests that immunotherapy might be useful for ALK-positive NSCLC patients who were treated with or without ALK-TKIs. Subsequently, the FDA granted approval to the anti-PD-1 agent nivolumab for the treatment of patients with metastatic squamous NSCLC with progression on or after platinum-based chemotherapy on March 4, 2015. PD-1 positive tumor infiltrating lymphocytes and PD-L1 expressing tumor cells were seen in 18 of 42 cases (43%) of which 8 cases lacked other biologic targets including EGFR mutations, HER2, cMET, ALK, or ROS1 rearrangements [74]. In addition, PD-L1 expression was high in NSCLC tumors with ALK translocations (*n* = 10) [75]. Studies on immune activation/T cell infiltration in response to ALK inhibitors will be needed in the future.

In conclusion, the mechanisms of acquired resistance to ALK inhibitors are just beginning to be understood. They should be exactly elucidated. New molecular-targeted drugs and their mechanisms of resistance must be understood in detail, which would allow the appropriate therapeutic strategies to be applied.
